# The 100 most influential articles in mouth breathing. A bibliometric and altmetric analysis: 2002-2021

**DOI:** 10.4317/medoral.26845

**Published:** 2024-11-25

**Authors:** Alicia Odette Campo, Pilar Valderrama, Manuel Bravo, Adela Baca

**Affiliations:** 1 Master postgraduate student. Faculty of Dentistry, University of Granada, Granada, Spain; 2 Postdoctoral Researcher. Department of Information and Communication, University of Granada, Granada, Spain.; 3 Professor, Department of Stomatology, Faculty of Dentistry, University of Granada, Granada, Spain; 4 Orthodontic Private Practice, Associate Professor, Department of Stomatology, Faculty of Dentistry, University of Granada, Granada, Spain

## Abstract

**Background:**

This study aimed to identify and analyze the most influential Mouth Breathing (MB) articles in children and adolescents with the highest relative citation rates (RCRs), through bibliometric and altmetric analysis, from 2002 to 2021.

**Material and Methods:**

On March 27, 2023 a PubMed search was conducted to detect papers published about MB. From a total of 826 documents, the article data were downloaded from iCite database. The 100 articles with the highest RCRs were selected for analysis in terms of RCR, citations, altmetric attention score, year, journal, first author (name, institution, country), subfield and design of study. The key words were analyzed using Vosviewer.

**Results:**

Among the 100 articles, there were no differences between the two periods analyzed for RCR and AAS values, yet 2002-11 was more cited than 2012-2021. There was no correlation between RCR and AAS; but there was with citations. Brazil was the most cited country (*n*=30). The articles were published in 48 journals pertaining to 8 categories, 44 corresponding to Dentistry. The most frequent study design was the cross-sectional (*n*=48). Although all subfields were well represented, the most frequent ones were “treatment”, “sleep disorders” and “clinical characteristics/cephalometry”. The most prominent keywords were “malocclusion” and “cephalometry”.

**Conclusions:**

Using RCR, a time- and field-normalized metric, one can identify influential articles in MB, a multidisciplinary research field of great importance for orthodontics. Because this bibliometric approach reduces the time from publication to the detection of an article´s importance for readers, it could be a valid alternative to using citation counts.

** Key words:**Bibliometrics, altmetrics, mouth breathing, children and adolescents.

## Introduction

Mouth Breathing (MB) is a very frequent habit among children and adolescents, and in many cases a disorder of the respiratory system that partly or fully replaces nasal breathing. Its etiology is complex and multifactorial, having an anatomical obstructive and/or functional origin (1).

Patients who breathe through their mouth run the risk of developing skeletal alterations of their facial features, malocclusions, problems with chewing, sleep disorders, poor life quality, cognitive deterioration, and postural problems with systemic repercussions (2-5). Even after the cause of the obstruction is eliminated, or the function is normalized, MB can continue because of habit. Due to its multidisciplinary nature, it is difficult to establish a standardized diagnosis of MB, and the application of a global clinical focus implies collaboration among different medical fields (6).

Bibliometrics is a quantitative analytical and statistical discipline focused on the analysis of research papers published in scientific journals by means of diverse metrics. Bibliometric tools help evaluate and filter various aspects of scientific articles, which makes it possible to highlight relevant data, areas, and trending topics, and the most cited or classic articles, as well as to view the evolution of research along more general lines (7).

The citation count is the index most commonly used to evaluate the quality of individual publications, but it entails certain limitations. Citations are highly dependent on academic areas, meaning there is a need for field normalization. Moreover, they are time-dependent —slow to reveal the scholarly use of articles and their overall implications— so that there is likewise a need for time normalization (8).

In a multidisciplinary field such as MB, in order to identify trends, a metric that is both time- and field-normalized, such as the Relative Citation Rate (RCR), may therefore prove relevant (9). It is a ratio of rates. The time normalization is calculated as the number of times a paper is cited divided by the number of years since its publication. To normalize the subfields, they are sampled for each article by means of its co-citation network. For instance, an RCR value of 1 indicates that a given article has been cited the same number of times per year (cites/year) as the mean for articles in that subfield and year. The RCR has been used, among other applications, to appraise a discipline´s most influential articles (10,11).

There is a growing awareness of the impact of research divulged by public platforms and social networks, suggesting an alternative means for appraising the influence of research, beyond metrics based on citation. Altmetric Attention Score (AAS) is an alternative metric determined by an automatized algorithm derived from mentions on several platforms or social networks. It considers their quality, so that mentions in national or international newspapers count more than the blog entries. The altmetric points help readers identify those articles generating most interest on the web (12). This has led to studies of traditional metrics as opposed to altmetrics, although results to date have been inconclusive or contradictory (13).

Globally, orthodontics has been the object of focused bibliometric studies, e.g. the most cited articles (14-17) or those with the highest AAS (18), including specific topics (19-22). Until now, no bibliometric analysis of MB has appeared published; some studies have looked at specific aspects that may be involved (23). Our intention was to identify and analyze the most influential MB articles in children and adolescents with the highest RCR, through bibliometric and altmetric analysis over the last two decades.

## Material and Methods

- Search strategy

On March 27, 2023, an advanced search was conducted, without language restriction, in PubMed. The strategy was as follows: ((breathing, mouth[MeSH Terms] OR "mouth breath*"[ Title/Abstract] OR "mouth breath*"[Text Word] OR "oral breath*"[Title/Abstract] OR “oral breath*”[Text Word]) AND ("child"[MeSH Terms] OR "child*"[Title/Abstract] OR "child, preschool"[MeSH Terms] OR "preschool children"[Title/Abstract] OR "Preschool"[Title/Abstract] OR "adolescent"[MeSH Terms] OR "adolescen*"[Title/Abstract] OR "teenager*"[Title/Abstract] OR "Youth"[Title/Abstract]) AND 2002/01/01:2021/12/31[Date - Publication]).

The number of articles recovered was 826; from PubMed, the PMID numbers of the articles were exported to iCite (9) to obtain the RCR and citation values. Nineteen were eliminated because their date of publication was after 2021, leaving 807 documents ordered according to their RCR (from highest to lowest). Two researchers (OC and AB) independently reviewed the abstracts and/or full texts to select those that addressed the topic of study, including only original articles and reviews. In case of doubt, a consensus was reached and the 100 articles with the highest values were selected. Furthermore, the AAS values were obtained from Almetrics.com. Studies about MB in children and adolescents (up to age 18) were included, whether daytime or nighttime MB. Seven articles involving adult patients were excluded, along with 42 not directly related to MB.

The following information was extracted from each article: title, author name(s), institution and country of origin of the first author, year and title of the journal. Classification by research subfield and study design was done by the same researchers, independently, after reading the titles, abstracts and/or full text. In the case of discrepancy, a consensus was reached.

- Analysis of keywords using VosViewer maps

To develop the keyword maps, the program Vosviewer 1.6.17 was used (Centre for Science and Technology Studies, Leiden University, The Netherlands; https://www.vosviewer.com) (24). The network was analyzed taking keywords with at least three co-occurrences. This program is very user-friendly, providing a visualization of bibliometric data, in which the size of the nodes reflects the frequency of the keywords used, while the thickness of the lines indicates the proximity of interactions between nodes. Colors are used to distinguish temporal lines.

- Statistical analysis

The Shapiro-Wilk test was used to determine if RCR, citations and AAS followed a normal distribution. The Mann-Whitney test served to compare metrics between the two decades, and the Kruskal-Wallis test was applied to compare the metrics between the subfields of study and the study designs. A two-way Pearson correlation was carried out for RCR, citations and AAS. Statistical analysis was done with the program SPSS, version 28, under license by the University of Granada. The level of significance was set at *p*<0.05.

## Results

- Bibliometrics

The 100 articles about MB having the highest RCR values for the period 2002-2021 are shown in Supplement 1, which includes the RCR value, range, citations and AAS. The RCR values ranged from 6.31 to 1.53. For the 100 articles of reference, citations ranged between 117 and 2. Out of the total (100), 34 articles were not registered in Altmetric.com, and six had a value of 0; 45 articles had an AAS between 1 and 9, and twelve had between 10 and 60 AAS. Three articles had an AAS value over 100. There was a significant correlation between RCR values and citations (*p*<0.001), but not between RCR and AAS (0.765) or citations and AAS (0.621).

**Figure 1:**
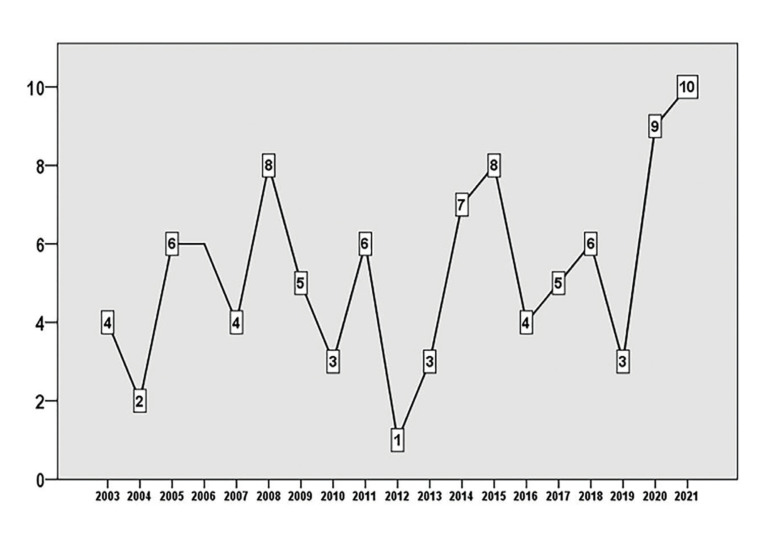
Number of articles published each year.

Except for 2002, we worked with articles published in all the years of the two decades studied. Fig. 1 shows the number of articles per year. Outstanding is the year 2021, with 10 articles; followed by 2020 with nine. Table 1 presents the bibliometric characteristics of the 100 articles by decade (2002-2011 and 2012-2021). Although the RCR and AAS values do not give significant differences between the two periods, for the citations the difference is statistically significant, the median being 50 for the first period and 17 for the second. Analysis of these metrics by year indicates the same behavior, without statistically significant differences, for RCR (*p*=0.427) and AAS (*p*=0.417), whereas for citations the difference is significant (*p*<0.001).

- Countries, institutions, journals and authors

Concerning the first author, a total of 22 countries contributed to the 100 articles studied (in view of their RCR). Outstanding is Brazil, with 30, then Italy (*n*=19), China (*n*=13), USA (*n*=10), Canada (*n*=4) and Spain (*n*=3). Germany, Israel, Slovenia, Taiwan and Turkey contributed with two publications each, and another 11 countries produced one document. A total of 65 institutions were represented in the 100 articles. Table 2 gathers those with at least two articles published, showing the country, the total RCR, citations, and AAS.

These 100 articles were published in 48 journals included under eight categories of the Journal Citation Reports. Altogether, 44 articles appeared in dentistry journals, and 56 in journals of other categories. International Journal of Pediatric 0torhinolaryngology gave the highest number of publications, of total RCR, and of citations (*n*=14, Weighted RCR 37.49, citations 695, and sum AAS 240) (Table 3). Twenty-two documents were published in journals specifically in the realm of Orthodontics highlighting, Angle Orthodontist, American Journal of Orthodontics and Dentofacial Orthopedics and European Journal of Orthodontics. Table 3 shows those with at least two articles. it is followed by (*n*=6, 15.51 total RCR, 267 citations, 9 AAS).

**Figure 2:**
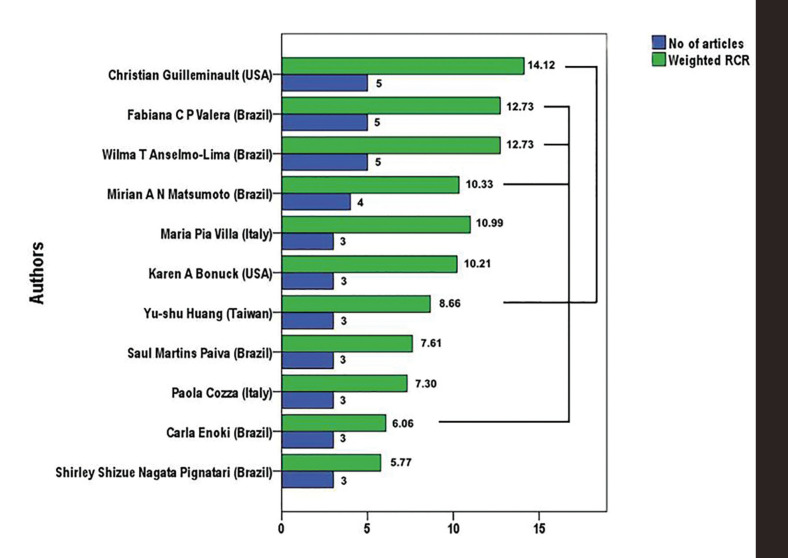
Authors of three or more articles among the 100 Mouth Breathing papers with the highest relative citation rates (RCR) in the period 2002-2021. Lines indicate co-authorship of articles.

A total of 462 authors contributed to the 100 articles having the highest RCR. The number of authors per article ranged between 1 and 12, with a mean of 5.39 (standard deviation 2.32) authors per article. Just three articles had only one undersigning author, while 35 had between two and four authors. Fig. 2 displays the number of articles and the total RCR of those authors undersigning three or more articles.

- Subfields of study, study design, and keywords

Table 4 indicates the frequency of the articles according to their subfield of study and study design, as well as the weighted RCR, total citations and AAS. The global comparison of these three variables did not give significant differences for any of the three metrics.

**Figure 3:**
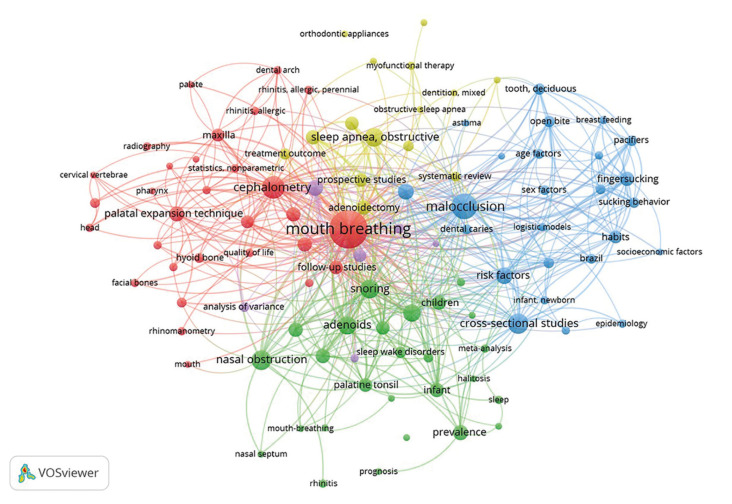
The network of three or more co-occurring keywords of the top RCR articles in Mouth Breathing: 2002-2021. A total of 102 nodes, five clusters, 1618 links, and a total link strength of 2828.

**Figure 4:**
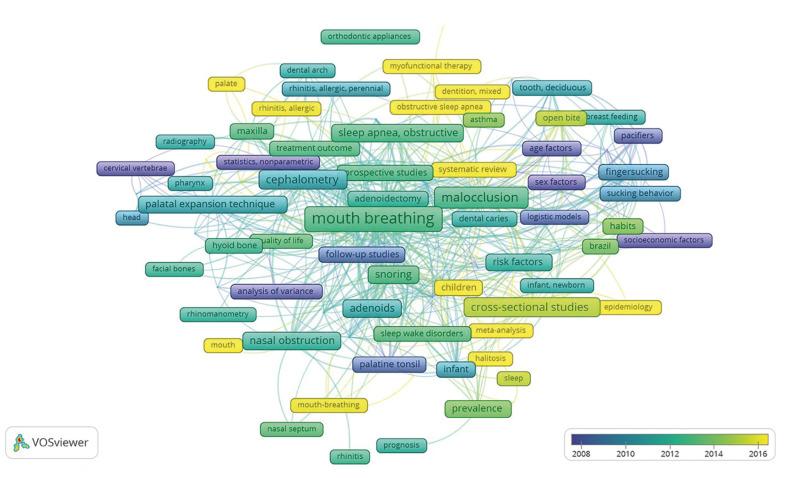
The network of three or more co-occurring keywords as a timeline of the top RCR articles in Mouth Breathing: 2002-2021. A total of 102 nodes, five clusters, 1618 links, and a total link strength of 2828. The color yellow represents the most recent keywords, while dark blue shows the oldest keywords.

From the top 100 RCR articles, a total of 378 keywords were obtained, and 102 met the threshold of having three or more co-occurrences (excluding “human”, “child”, “male”, “female”, “child, preschool”, “adolescent” and “adult”), with 5 clusters, 1618 links, and a 2828 total link strength (Fig. 3). The most frequent keywords were “mouth breathing” (n = 59), “malocclusion” (n = 29), “cephalometry” (n = 23), “cross-sectional studies” (*n*=19), “nasal obstruction” (*n*=18), “snoring” and “sleep apnea, obstructive” (*n*=17), “adenoids” (*n*=16), “surveys and questionnaires” (*n*=14), “risk factors” (n = 13), mandible, case-control studies (*n*=12) and palatal expansion technique (*n*=11). The network timeline of at least three co-occurrences is shown in Fig. 4.

## Discussion

Bibliometric and altmetric analysis could be useful for identifying interesting or hot topics in the research realm and help clarify the current scientific outlook regarding MB for health professionals involved in its diagnosis and treatment. This article is based on the identification and bibliometric analysis of the 100 MB-related publications having the highest RCR, including in the evaluation citations and AAS values. The time period studied was 20 years (2002-2021); 2022 was not included because the RCR values of the previous year are provisional, calling for some exposition time to analyze AAS (25). After 2002 the data were available on Google and Wikipedia platforms, making it possible to analyze AAS, as the values are based on participation in social networks. PubMed was used because it is largely focused on medicine and biomedical sciences, it is of open access worldwide (26), and the RCR values are available for free through iCite (https: //icite.od.nih .gov/).

The citation count is the index most commonly used to assess the quality of articles, but it is important to stress that citations are highly dependent on academic fields and time of publication (8). For this reason, the indicator RCR was selected. Identifying trends calls for the inclusion of articles that are recent and have not had much time to be cited enough to appear on the lists of most-cited articles. A paper may need two years to appear on the lists (21), or four years (14), or even seven years (16). Regarding this indicator, 19% of the articles dealt with here were published in 2020-2021.

Analysis of the metrics showed no significant difference between the two periods studied in terms of RCR, which would confirm that it is a time-normalized indicator. In contrast, the number of citations is significantly higher in the older decade, because citations accumulate over time. A correlation was found between RCR and citations, but not between RCR and AAS; this is not unusual (18,27) and suggests that both metrics be viewed as complementary; it is accepted that no single metric is sufficient to identify articles of scientific interest (28).

The most influential article, with an RCR of 6.31 and 9 cites, was published by Zhao Z *et al* (2021) (1). It is a systematic review and meta-analysis of clinical trials and cohort studies, evaluating the effects of MB on facial skeletal development in children, receiving much attention on the web, with an AAS of 60. The second, by D'Onofrio L (2019) (29), is a narrative review (RCR 6.29, cites=25 and AAS=6) about how diverse oral dysfunctions, among them MB, impact occlusal and facial development. The third most influential study is by Harari D *et al* (2010) (30) (RCR 4.82, cites=92 and AAS=36), a retrospective study whose objective was to determine the effect of MB during childhood on craniofacial and dentofacial development compared to nasal breathing in malocclusion patients treated in orthodontic clinics. The fourth (RCR 4.75 and AAS=1) obtained the highest number of cites (*n*=117) and was published by Valera FC *et al* (2003) (31), a group of very well ranked authors (Fig. 2) from the institution best represented: University of São Paulo (Brazil). Its aim was to characterize dentofacial alterations that occur in children with hypertrophy of the adenoids, alone or associated with hypertrophy of the palatine tonsils.

Despite the leadership of USA in orthodontic research (16,8,19), including randomized controlled trials (32), Brazil stands out with 30/100 articles. Indeed, there is a growth of interest in quality research in this field among countries of Asia and South America (14,21). Brazil is making noteworthy progress in biomedical research and in Orthodontics (33). In specific related topics such as artificial intelligence (22) or lingual orthodontics (18), South Korea and Germany respectively rank very high. The outstanding institutions are Brazil´s Federal University of São Paulo, University of São Paulo and Federal University of Minas Gerais, producing 19 articles. In fifth place we find Stanford University, USA, no doubt because the most prolific author is rooted there: Christian Guilleminault is a world reference, working on sleep disorders associated with breathing, the first researcher to describe Obstructive Apnea in children´s sleep problems, and the role of the dentist in multidisciplinary treatment.

Out of the 48 journals publishing the 100 articles studied here, 22 articles were published in six journals specifically dedicated to orthodontics, making manifest the relevance of this area in the multidisciplinary treatment of pathologies deriving from MB. Keyword analysis revealed the strong association between MB and malocclusion and cephalometry, both closely related with orthodontics; the size of the keyword nodes reflects that they are two of the terms most frequently used in the research articles analyzed (Fig. 3). According to Journal Citation Reports, the journals belong to eight categories (Table 3), their positions varying, depending on the Aggregate Impact Factor (AIF) —-similar to the JIF but taking into account all the journals in the category. This finding would confirm that MB is multidisciplinary and supports the use of a field-normalized indicator to avoid skipping over articles published in lower-ranking categories such as Otorhinolaryngology (with an AIF of 2.4) or Dentistry (3.2) as opposed to Critical Care Medicine (AIF=7.3).

The articles showed heterogeneity in subfields of study and study design, yet no statistically significant differences in RCR values, citations and altmetrics. Regarding the subfields of study, we encountered influential articles in all categories, the three most prolific ones being “treatment”, “sleep disorders” and “clinical characteristics/cephalometry”. It is interesting to note that there are no significant differences among the six subfields in RCR, citations or AAS. This could mean that each one constitutes an important area that is well addressed in MB studies.

With respect to the study design, original articles make up 82% of the output, led by cross-sectional (48/100) and cohort studies (21/100). This has been previously reported for bibliometric studies on orthodontics (16) and bruxism (22). The least frequent are experimental studies that include randomized clinical trials, most likely because they are difficult to carry out in pediatric populations and would be costly (34). The systematic review/meta-analysis and narrative review made up 18% of the total, although the former are very relevant in terms of scientific evidence and usually highly cited, which might have led to an overproduction of this study design (35), hence less citation.

Fig. 4 shows the most recent keywords in yellow. Two of the salient areas, marking current trends, are “obstructive sleep apnea” (AOS) and “myofunctional therapy”. The diagnosis and treatment of AOS can be very complex, entailing a multidisciplinary approach by otorhinolaryngologists, surgeons and orthodontists (17). This “overlap” of specialties would mean increased citation and interest in the related disorders and pathologies, even after the origin of the problem is eliminated (36). Respiratory disorders in the sleep of children and adolescents, such as AOS or MB, may give rise to a muscular dysfunction, alterations in maxillary growth and development, or malocclusions, treated through orthodontics combined with functional apparatuses to re-establish and normalize orofacial function. Recent bibliometric studies in the field of orthodontics highlight work that evaluates the myofunctional therapies and new techniques for upper maxillary expansion needed for many cases of young patients with breathing disorders (16,21). Halitosis is another noteworthy keyword. Deserving mention is the article with the highest AAS (RCR= 2.53, 41 cites and 303 AAS) (37), focusing on halitosis in association with MB. The social and psychological repercussions of halitosis make it a trending topic (hence salient node), of widespread interest and concern, implying a strong need to acquire quality information thereabouts. This would explain the high AAS score.

Interpretation of the results expounded here calls for some consideration of the study´s limitations. First, there may have been a bias related to the presence of self-citation, or the potential preference of some authors to cite articles from a specific journal. In addition, the articles were identified only from PubMed, and the methodological quality of the studies included was not appraised.

## Conclusions

The use of a normalized (over both time and field) metric such as RCR to identify the 100 most influential articles about MB in children and adolescents allowed us to recover recent publications, thereby permitting the identification of research trends. The articles were published in 48 scientific journals of diverse categories, confirming that MB constitutes a multidisciplinary field. Brazil stands out at the forefront of related output.

The 100 articles with the highest RCR mostly dealt with the subfields “treatment”, “sleep disorders” and “clinical characteristics/cephalometry”, and the most frequent design was the transversal study.

The combination of metrics based on citation (RCR and cites) and alternative metrics (AAS), as complementary means, may help illustrate a more representative research panorama.

## Figures and Tables

**Table 1:** Metrics of the top 100 most influential Mouth Breathing articles with the highest relative citation rates (RCR): 2002 - 2021.

Metrics		2002-2021	2002-2011	2012-2021	Comparison p value*
RCRs	n	n=100	n=44	n=56	0.429
	RCRs^a^	2.28 (1.31)	2.3 (1.41)	2.27 (1.2)	
	Min-Max	1.53-6.31	1,57-4.82	1.53-6.31	
	Weighted RCR	262.93	117.36	145.58	
Cites	n	n=100	n=44	n=56	<0.001
	Cites^a^	33.5 (37)	50 (35)	17 (19)	
	Min-Max	2-117	27-117	2-80	
	Total citations	3722	2610	1112	
AAS	n	n=66	n=24	n=42	0.211
	AAS^a^	3.5 (8)	3 (6.75)	5 (8.75)	
	Min-Max	0-303	0-303	0-192	
	Total AAS	1183	615	568	

^a ^Median (interquartile range). Min - Max: Minimum and Maximum values. Weighted RCR: sum of the RCRs for the articles in the group.^ *^Mann-Whitney test, previously the Shapiro-Wilks test showed no normality. Altmetric Attention Score (AAS).

**Table 2:** Institutions that contributed at least two articles to the top 100 most influential Mouth Breathing articles with the highest relative citation rates (RCRs): 2002 - 2021.

Rank	Institutions** (Countries)	Country	No. articles	Sum*		
				RCR	Citations	AAS
1	Universidade Federal de São Paulo	Brazil	7	14.67	164	75
2	University of São Paulo (FORP-USP)	Brazil	6	16.48	348	4
3	Federal University of Minas Gerais	Brazil	6	14.91	152	18
4	"Sapienza" University, Rome	Italy	5	14.86	188	23
5	Stanford University Sleep Medicine Division	USA	4	12.21	249	10
6	The University of Hong Kong	China	3	7.10	205	2
7	Albert Einstein College of Medicine	USA	2	8.68	175	218
8	Catholic University of Sacred Heart, Dental Institute	Italy	2	8.23	61	23
9	Universidad de Sevilla	Spain	2	6.59	67	4
10	University of Rome "Tor Vergata"	Italy	2	5.66	61	1
11	Imola Hospital, Imola	Italy	2	4.07	87	-
12	State University of Campinas	Brazil	2	3.86	77	-
13	University G D'Annunzio, Chieti, Pescara	Italy	2	3.83	80	-
14	Beijing Children's Hospital, Capital Medical University	China	2	3.75	57	0
15	University of Ljubljana	Slovenia	2	3.74	77	14
16	Chung Chan Memory Hospital and University	China	2	3.56	59	-

*Sum of the RCRs, cites, and AAS for the articles of each institution. ** A total of 65 institutions contributes to the top 100 RCR articles. Institutions with the same number of articles were ordered according to their weighted RCR. Altmetric Attention Score (AAS).

**Table 3:** Journals that contributed at least two articles to the top 100 most influential Mouth Breathing articles with the highest relative citation rates (RCRs): 2002 - 2021.

Rank	Journal**	No	Categories (JCR)	Q	JIF 2022	Weighted RCR^*^	Sum*	
							Cites	AAS
1	International Journal of Pediatric 0torhinolaryngology	14	Otorhinolaryngology Pediatrics	Q3 Q4	1.5	37.49	695	240
2	Angle Orthodontist	6	Dentistry	Q2	3.4	15.51	267	9
3	Sleep Medicine	5	Clinical neurology	Q1	4.8	11,09	102	2
4	Chest	4	Respiratory System Critical Care Medicine	Q1 Q1	10.1	11.67	288	10
5	American Journal of Orthodontics and Dentofacial Orthopedics	4	Dentistry	Q2	3	11.41	213	8
6	European Journal of Orthodontics	4	Dentistry	Q3	2.6	10,70	206	18
7	Sleep And Breathing	3	Clinical neurology Respiratory System	Q3 Q3	2.5	12.27	149	23
8	Progress In Orthodontics	3	Dentistry	Q1	4.8	5.99	50	3
9	Dental Press Journal of Orthodontics	3	NO JCR	-	-	5.65	53	9
10	International Journal of Paediatric Dentistry	3	Dentistry Pediatrics	Q1 Q1	3.8	5,93	120	6
11	BMC Oral Health	2	Dentistry	Q2	2.9	9,49	46	65
12	Orthodontics & Craniofacial Research	2	Dentistry	Q2	3.1	8,17	34	11
13	Acta Otorhinolaryngologica Italica	2	Otorhinolaryngology	Q3	2	7,13	48	23
14	Sleep	2	Clinical neurology Neurosciences	Q1 Q1	5.6	6,69	152	26
15	Jornal De Pediatria	2	Pediatrics	Q2	3.3	6.45	75	9
16	Pediatrics	2	Pediatrics	Q1	8	5,90	131	192
17	European Journal of Paediatric Dentistry	2	Dentistry Pediatrics	Q1 Q1	3.6	5,34	23	1
18	Journal of Clinical Pediatric Dentistry	2	Dentistry Pediatrics	Q4 Q4	1.3	4.64	39	4
19	Journal of Otolaryngology-Head & Neck Surgery	2	Otorhinolaryngology	Q1	3.4	4,63	25	37
20	Pediatric Pulmonology	2	Pediatrics Respiratory System	Q2 Q3	3.1	4.37	133	1
21	Cochrane Database of Systematic Reviews	2	Medicine, General & Internal	Q1	8.4	4.33	60	71
22	Brazilian Journal of Otorhinolaryngology	2	Otorhinolaryngology	Q2	2.2	3,96	51	1
23	CRANIO-The Journal of Craniomandibular & Sleep Practice	2	Dentistry	Q4	1.6	3.79	43	1

*Weighted RCR and sum cites or AAS: Sum of the RCRs, cites, or AAS for the articles of each journal. ** A total of 48 Journals contributes to the top 100 RCR articles. Journals with the same number of articles were ordered according to their weighted RCR. Dentistry is the category "Dentistry, Oral surgery and Medicine", JCR: Journal Citation Report, JIF: Journal impact factor; Q: quartile according to JIF 2022.

**Table 4:** Field of study and study design of the top 100 most influential articles in Mouth Breathing (MB) by the highest relative citation rates (RCR): 2002-2021.

Field of study and Study design		No	Weighted RCR (mean/article)	Sum cites (mean/article)	No	Sum AAS (mean/article)
Field of study	Risk factors/etiology	18	44.65 (2.48)	571 (31.72)	13	91 (7)
	Sleep disorders	20	54.29 (2.71)	923 (46.15)	14	251 (17.93)
	Malocclusion	14	43.88 (3.13)	478 (34.14)	11	58 (5.27)
	Clinical characteristics/cephalometry	20	51.40 (2.57)	634 (31.7)	14	672 (48)
	Treatment	21	50.74 (2.42)	830 (39.52)	11	96 (8.72)
	MB as clinical characteristic/ syndrome	7	17.97 (2.57)	286 (40,84)	3	15 (5)
	Comparison, p value*	-	0.261	0.341	-	0.465
Study design	Systematic review / Meta-analysis	12	31.68 (2.64)	336 (28)	8	119 (14.87)
	Clinical experimental study	6	12.95 (2.16)	186 (31)	3	2 (0.67)
	Cohort	21	51.18 (2.43)	900 (42.86)	19	303 (15.95)
	Case-control	7	18.82 (2.69)	266 (38)	5	218 (43.6)
	Cross-sectional	48	130.36 (2.71)	1800 (37.5)	28	528 (18.85)
	Narrative review/case description	6	17.36 (2.89)	234 (39)	3	4.33 (13)
	Comparison, p value*	-	0.684	0.666	-	0.347

Weighted RCR: sum of RCR values of the articles of each research subfield. *Global comparison by the Kruskal-Wallis test. The values did not show a normal distribution after the Shapiro-Wilk test.
